# The importance of mechanical constraints for proper polarization and psuedo-cleavage furrow generation in the early *Caenorhabditis elegans* embryo

**DOI:** 10.1371/journal.pcbi.1006294

**Published:** 2018-07-09

**Authors:** Betül Senay Aras, Y. C. Zhou, Adriana Dawes, Ching-Shan Chou

**Affiliations:** 1 Department of Mathematics, The Ohio State University, Columbus, Ohio, United States of America; 2 Department of Mathematics, Colorado State University, Fort Collins, Colorado, United States of America; 3 Department of Molecular Genetics, The Ohio State University, Columbus, Ohio, United States of America; Centrum Wiskunde & Informatica (CWI) & Netherlands Institute for Systems Biology, NETHERLANDS

## Abstract

Intracellular polarization, where a cell specifies a spatial axis by segregation of specific factors, is a fundamental biological process. In the early embryo of the nematode worm *Caenorhabditis elegans (C. elegans)*, polarization is often accompanied by deformations of the cortex, a highly contractile structure consisting of actin filaments cross-linked by the motor protein myosin (actomyosin). It has been suggested that the eggshell surrounding the early embryo plays a role in polarization although its function is not understood. Here we develop a mathematical model which couples a reaction-diffusion model of actomyosin dynamics with a phase field model of the cell cortex to implicitly track cell shape changes in the early *C. elegans* embryo. We investigate the potential rigidity effect of the geometric constraint imposed by the presence and size of the eggshell on polarization dynamics. Our model suggests that the geometric constraint of the eggshell is essential for proper polarization and the size of the eggshell also affects the dynamics of polarization. Therefore, we conclude that geometric constraint on a cell might affect the dynamics of a biochemical process.

## Author summary

Polarization, whereby molecules and proteins are asymmetrically distributed throughout the cell, is a vital process for many cellular functions. In the early *C. elegans* embryo the asymmetric distribution of cell cytoskeleton during the initiation of polarization leads to asymmetric contractions which are higher in the anterior and lower in the posterior of a cell. The *C. elegans* embryo is surrounded by a rigid body, the eggshell, which functions in numerous cell processes. We investigate the structural support of eggshell during the establishment phase by tracking the moving cell surface. We incorporate protein dynamics involved in polarization into the membrane evolution. We conclude that eggshell might have a role in cell polarization by preventing the distortion of cell surface.

## Introduction

The geometry of a cell can have a profound influence on cell function and survival [1–4]. Cell geometry is generated by internal structures such as the cytoskeleton, and can also be externally imposed by interactions with neighboring cells or mechanical structures like an eggshell. Eggshells are critical for early development of the nematode worm *C. elegans* as it prevents multiple sperm from fertilizing a single egg, along with ensuring proper chromosome segregation during meiosis, and proper organization of membrane and cortical proteins [5–7].

Shortly after fertilization, the *C. elegans* embryo polarizes by asymmetrically localizing specific proteins, including actin, myosin and polarity determinants such as the Par proteins, in response to a cue from the sperm [8, 9]. Polarization of the embryo proceeds in two distinct phases: establishment and maintenance [10]. During the establishment phase, the developmental time frame we consider in this paper, the actomyosin cortex, a thin structure below the membrane consisting primarily of polymerized actin filaments and cross-linked by the motor protein myosin, is highly dynamic and contractile, creating small invaginations on the cell surface called ruffles [9, 11]. The cue locally relaxes the actomyosin cortex causing local loss of ruffles and initiation of cortical flow that transports the anterior Par proteins, PAR-3, PAR-6 and aPKC, towards the anterior pole. This allows the posterior Par proteins, PAR-1, PAR-2 and LGL, which are mutually antagonistic to the anterior Par proteins, to bind to the cleared area at the posterior pole [10, 12, 13]. During advection of the Par proteins and the actomyosin cap, a domain of high actomyosin density, towards the anterior pole, an invagination similar to the ruffles but much deeper, called the pseudocleavage furrow, forms and moves with the edge of the actomyosin cap and the interface between the anterior and posterior Par proteins [9, 10, 14]. At the end of the establishment phase, the pseudocleavage furrow along with the actomyosin cap and the Par protein interface reach the middle of the cell. The pseudocleavage furrow retracts and the segregated Par protein domains are held through the maintenance phase as the cell prepares for first division.

It has been demonstrated that the pseudocleavage furrow and ruffles are not essential for proper polarization. A maternal effect mutation *nop-1*(it142) eliminates cortical contractility during the establishment phase which results in the absence of the pseudocleavage furrow and ruffles but does not appear to interfere with proper development despite attenuation of actomyosin asymmetry and cortical flow [15, 16]. Although it is known that the pseudocleavage furrow and ruffles are generated as a result of contractility, and the pseudocleavage furrow appears at the boundary between high and low actomyosin concentrations, the mechanical generation of these invaginations is not fully understood.

The developing *C. elegans* embryo is surrounded by an eggshell, a rigid body containing the protein chitin, which is created by the embryo after fertilization and persists until hatching. When the eggshell is genetically or chemically manipulated prior to the two cell stage, the cells are highly mechanically sensitive and fail to properly extrude polar bodies at the beginning of the establishment phase, and do not properly polarize or form the pseudocleavage furrow [5–7]. Removal of the eggshell at or after the two cell stage does not interfere with development [17]. Since chitin is responsible for eggshell rigidity [5], this suggests that the eggshell may provide structural support during the asymmetric actomyosin contractions associated with polarization [5], although this role is not well understood.

In this paper, we develop a mathematical model to investigate the mechanical effect of the eggshell during establishment of polarization. This model allows us to modify or remove the eggshell completely to explore the role of the eggshell shape and rigidity on polarization. The model simulates the morphological changes of the membrane during establishment of polarization which includes movement of the pseudocleavage furrow. Reaction-diffusion equations describing the dynamics of Par proteins and actomyosin [1] are incorporated into the moving interface problem. Cell shape dynamics are tracked using the phase field method which is widely used for moving interface problems [18–20].

In the phase field method, the interface is not explicitly tracked, which avoids potential numerical difficulties associated with the classical Lagrangian method. Here the interface is the level set of an auxiliary field *ϕ*. *ϕ* takes on different constant values corresponding to inside and outside of the cell and these constant values are connected by a smooth interface. In our model, cortex is assumed to be a viscous material and the asymmetric actomyosin contractions in the cortex drives cell deformations. The interactions between membrane bending energy, surface tension, eggshell constraint and forces applied by the cortex determines the current state of the cell. Using numerical simulations we found that the eggshell rigidly supports the membrane and reinforces the polarization process. We also showed the importance of the size of the eggshell for proper polarization. Our results suggest that mechanical constraints on cells imposed by structures such as an eggshell might affect dynamics and localization of proteins.

## Materials and methods

### Mathematical model

In the previous work by Dawes et al. [1], a model of reaction-diffusion equations was developed to capture the dynamics of Par proteins and actomyosin on a fixed domain. In their model, cortical tension is assumed to be linearly proportional to actomyosin concentration and the advective speed of protein movement is linearly proportional to the gradient of cortical tension. The solution is shown to have a moving interface between high and low concentrations of the proteins, which represents the movement of the interface between the anterior and posterior domains during the protein movement.

While the model in [1] is capable of capturing the essential features of the protein dynamics with relatively simple equations, it does not include any change in cell shape and therefore is limited in providing information about the physics of the entire polarization process. Here we consider the same species and reactions, and along with protein dynamics, we include the formation and movement of the pseudocleavage furrow as well as the constraint of the eggshell. In our model, since the plasma membrane and cortex are mostly in contact and deforming together, we do not distinguish the plasma membrane and cortex but assume they are a single structure, and in the following we use membrane or cortex to refer to this structure.

Following the model in [1], we do not consider individual Par proteins but group these proteins into two modules by their localizations: the anterior and posterior Par proteins. The anterior Par protein module consists of PAR-3, PAR-6 and aPKC, and the posterior Par protein module consists of PAR-1, PAR-2 and LGL. Thus, the model contains the following species with the corresponding notations:

*A*_*m*_: Cortical anterior Par protein monomers,
*A*_*sd*_: Cortical anterior singly bound dimers,
*A*_*dd*_: Cortical anterior Par protein doubly bound dimers,
*P*: Cortical posterior Par proteins,
*M*: Cortical actomyosin,
*A*_*y*_: Cytoplasmic anterior Par protein monomers,
*A*_2*y*_: Cytoplasmic anterior Par protein dimers,
*P*_*y*_: Cytoplasmic posterior Par proteins
*M*_*y*_: Cytoplasmic actomyosin.

We make the following key assumptions about the interactions as in [1, 21]:

- The anterior Par proteins can dimerize and bind to the cortex, and the anterior and posterior Par proteins promote each other’s dissociation from the cortex via phosphorylation.
- The interactions between these species follow mass-action kinetics.
- Cytoplasmic Par proteins, *A*_*y*_, *A*_2*y*_ and *P*_*y*_, are assumed to be at quasi-steady state and can associate with the cortex, and proteins dissociated from the cortex return to the cytoplasmic pool.
- Cytoplasmic actomyosin *M*_*y*_ is assumed to be at quasi-steady state. Cytoplasmic actomyosin assembles into the cortex and this assembly is negatively regulated by the posterior Par proteins.

**Fig 1:**
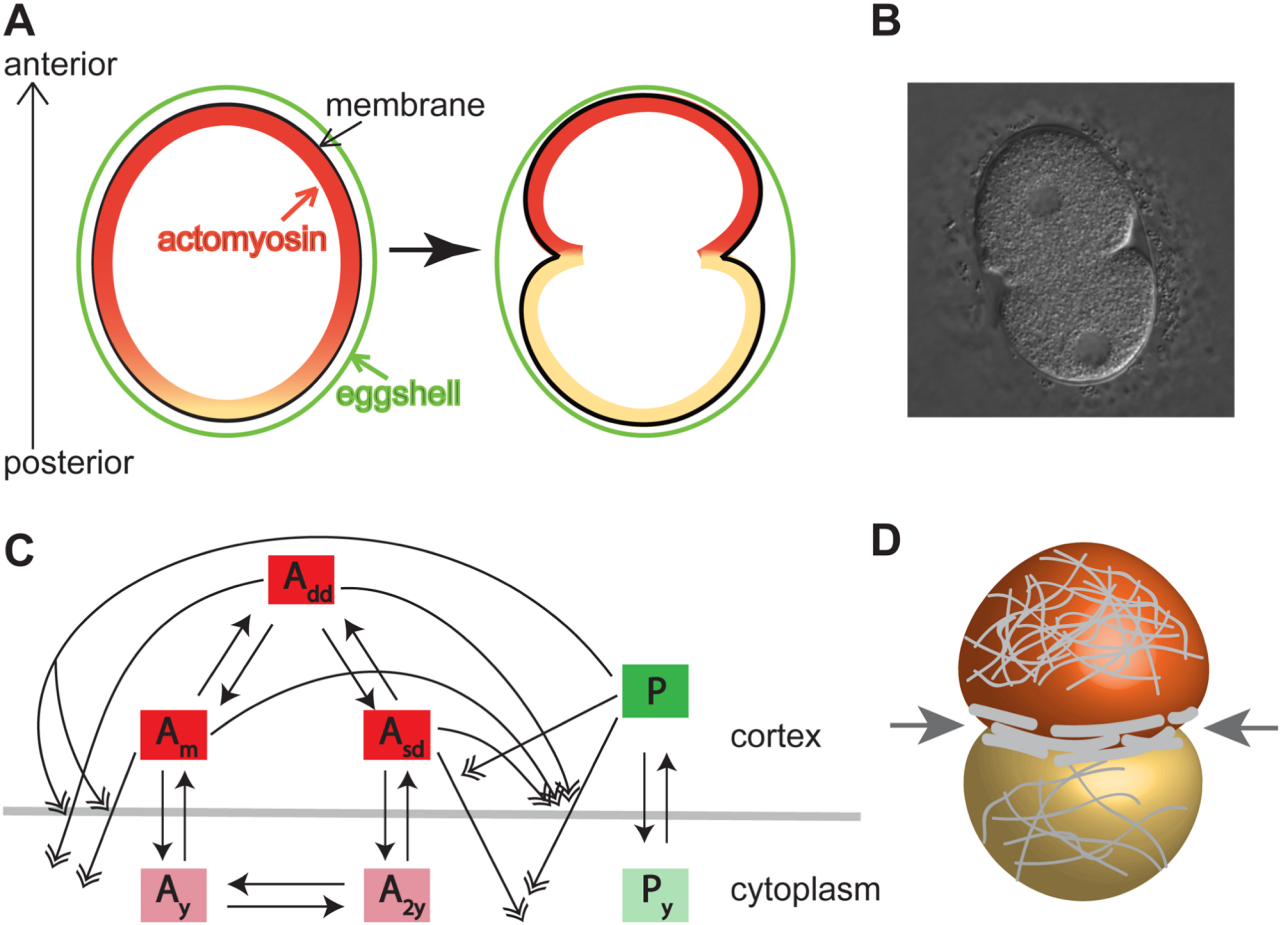
Illustration of the formation of pseudocleavage furrow, the cell geometry and simplified interactions between the Par proteins. (A) Actomyosin initially starts to retract to the anterior of the cell and the pseudocleavage furrow begins to form; later the invagination becomes deeper and moves towards to anterior pole; the color represents the concentration of actomyosin (yellow to red refers to low to high concentration). In this and all subsequent figures, the posterior pole is at the bottom and the anterior pole is at the top. (B) A DIC image of a typical wild type *C. elegans* embryo at the end of the establishment phase, when the pseudocleavage furrow has reached the middle of the cell. (C) Schematic of a simplified network based on experimentally determined interactions. *A*_*m*_: cortical anterior Par protein monomers, *A*_*sd*_: cortical anterior singly bound dimers, *A*_*dd*_: cortical anterior Par protein doubly bound dimers, *P*: cortical posterior Par proteins, *M*: cortical actomyosin. Solid arrowheads are cortical loss by conversion; double open arrowheads are cortical loss by regulated dissociation; light color species are phosphorylated forms. This figure is a reproduction of Figure 1 in [21]. (D) Illustration of the arrangement of actomyosin in a 3D view. The actomyosin proteins (gray curves on the membrane) are cross-linked away from pseudocleavage furrow and they are compressed in the furrow region due to the flow velocity gradient, leading to their alignment in the circumferential direction. The arrow indicates the inward force due to the actomyosin alignment.

The interactions between these species take place on the cortex, and therefore the domain of interest is the evolving cortex. A schematic diagram of the protein interactions are depicted in Fig 1C, and the protein dynamics can be described by the following equations:

$$\begin{array}{ccc} \frac{\partial [A_{m}]}{\partial t} & = & D_{pa}\nabla_{c}^{2}\left[A_{m}\right]-\nabla_{c}\cdot \left(v_{c}\left[A_{m}\right]\right)+k_{on}^{A}A_{y}-k_{off}^{A}\left[A_{m}\right]-2k_{d}^{+}{\left[A_{m}\right]}^{2}+2k_{d}^{-}\left[A_{dd}\right]\\ & & -k_{d}^{+}A_{y}\left[A_{m}\right]+k_{d}^{-}\left[A_{sd}\right]-r_{A}\left[P\right]\left[A_{m}\right], \end{array}$$ (1)

$$\begin{array}{ccc} \frac{\partial [A_{sd}]}{\partial t} & = & D_{pa}\nabla_{c}^{2}\left[A_{sd}\right]-\nabla_{c}\cdot \left(v_{c}\left[A_{sd}\right]\right)+k_{on}^{A_{sd}}A_{2y}-k_{off}^{A}\left[A_{sd}\right]+k_{d}^{+}A_{y}\left[A_{m}\right]-k_{d}^{-}\left[A_{sd}\right]\\ & & -k_{on}^{A_{dd}}\left[A_{sd}\right]+k_{off}^{A}\left[A_{dd}\right]-r_{A}\left[P\right]\left[A_{sd}\right], \end{array}$$ (2)

$$\begin{array}{ccc} \frac{\partial [A_{dd}]}{\partial t} & = & D_{pa}\nabla_{c}^{2}\left[A_{dd}\right]-\nabla_{c}\cdot \left(v_{c}\left[A_{dd}\right]\right)+k_{d}^{+}{\left[A_{m}\right]}^{2}-k_{d}^{-}\left[A_{dd}\right]-k_{off}^{A}\left[A_{dd}\right]+k_{on}^{A_{dd}}\left[A_{sd}\right]\\ & & -2r_{A}\left[P\right]\left[A_{dd}\right], \end{array}$$ (3)

$$\begin{array}{ccc} \frac{\partial [P]}{\partial t} & = & D_{pp}\nabla_{c}^{2}\left[P\right]-\nabla_{c}\cdot \left(v_{c}\left[P\right]\right)+k_{on}^{P}P_{y}-k_{off}^{P}\left[P\right]-r_{P}(\left[A_{m}\right]+\left[A_{sd}\right]\\ & & +2\left[A_{dd}\right])\left[P\right], \end{array}$$ (4)

$$\begin{array}{ccc} \frac{\partial [M]}{\partial t} & = & D_{m}\nabla_{c}^{2}\left[M\right]-\nabla_{c}\cdot \left(v_{c}\left[M\right]\right)+k_{on}^{M}M_{y}\frac{k_{P}}{k_{P}+\left[P\right]}-k_{off}^{M}\left[M\right]. \end{array}$$ (5)

In Eqs (1)–(5), $\nabla_{c}^{2}$ stands for the Laplace-Beltrami operator and ∇_*c*_ is the gradient operator on the membrane. The anterior Par proteins, posterior Par proteins and actomyosin diffuse on the cortex at the rates *D*_*pa*_, *D*_*pp*_ and *D*_*m*_, respectively. The protein movement induced by the initial cue which relaxes the cortex at the posterior pole drives the advection of anterior and posterior Par proteins. The advective velocities for all of the proteins, denoted by **v**_*c*_, are assumed to be linearly proportional to the gradient of cortical tension [1], which we assume to depend linearly on the actomyosin concentration, that is, **v**_*c*_ = *ν*∇*M* with *ν* a constant.

The Par proteins move along with the cortex, and therefore the aforementioned protein dynamics occurs on a deforming interface. To model the evolution of the cortex, we use the phase field model which implicitly tracks moving interfaces. The choice of phase field model over other explicit interface tracking methods, such as a Lagrangian frame, is based on the ease of coupling the protein dynamics with the deforming cortex, while avoiding numerical difficulties that may arise due to the deep invagination of the pseudocleavage furrow.

Since we are interested in the dynamics at the cell cortex, we use a 2D cross section of the cell as our domain $\Omega \subset R^{2}$, as shown in Fig 1A. We believe that this model, albeit simplified in geometry, will provide information on the possible mechanisms underlying cell-shape changes and pseudocleavage furrow formation. The phase field function *ϕ*(**x**) is defined on the entire domain *Ω*, where the region with *ϕ* = 0 represents the exterior of the cell, the region with *ϕ* = 1 represents the interior of the cell, and the region with 0 < *ϕ* < 1 is the “diffuse interface” including the level set *ϕ* = 0.5 that represents the cell membrane *Ω*_*s*_. The width of this diffuse interface is controlled by the transition parameter *ϵ*, which is taken to be a very small number. Note that the choice of *ϵ* depends on the spatial resolution: the spatial grid needs to be fine enough to resolve the diffuse interface. The evolution of the diffuse interface is governed by kinetic equations that is defined on the whole domain, and they will be described in the remainder of this section. Given a fixed membrane profile (i.e., a fixed *ϕ*), one can easily simulate the protein dynamics of Eqs (1)–(5) along the cell membrane by introducing *G*(*ϕ*) = 18*ϕ*^2^(*ϕ* − 1)^2^ in the equations. Note that *G*(*ϕ*), the double well potential, is nonzero only around the membrane, and therefore by multiplying *G* with the unknown variables or reaction terms, the protein dynamics are restricted to the neighborhood of the interface. In our numerical simulations, a non-dimensionalized version of model (1)–(5) was used. The non-dimensionalized model equations, coupled with *G*(*ϕ*), are displayed in Eqs. (S1)-(S5) of S1 Text.

In our model, the cell shape, i.e., the phase field function *ϕ*, is determined by the interactions between 1) the membrane bending energy, 2) membrane surface tension, 3) volume conservation, 4) eggshell constraint and 5) the force resulting from myosin contractility in aligned and cross-linked forms. Here we assume that that the cortex and cytoplasm are viscous with different viscosities, which is appropriate for long time scales [22–24]. The phase field function *ϕ* is governed by the following equation [19, 25]:

$$\begin{array}{c} \frac{\partial \phi }{\partial t}+u\cdot \nabla \phi =\Gamma (\epsilon \nabla^{2}\phi -G^{\prime }\left(\phi \right)/\epsilon +c\epsilon |\nabla \phi |), \end{array}$$ (6)

where **u** is the velocity field driving the membrane-cortex deformation, Γ the relaxation coefficient and *c* = ∇ ⋅ (∇*ϕ*/|∇*ϕ*|) the local interface curvature, which is added to stabilize the phase-field interface [19], *ϵ*∇^2^ *ϕ* − *G*′(*ϕ*)/*ϵ* is boundary free energy, which guarantees the existence of two phases connected through a smooth interface. The velocity field **u** is generated by the forces on the membrane, and it is also associated with the viscosity of the cortex and cytoplasm. Assuming that viscosity forces dominate advective inertial forces [23], **u** is the solution of the Stokes equation:

$$\begin{array}{c} \nabla \cdot \left[\eta \left(\phi \right)\left(\nabla u+\nabla u^{T}\right)\right]-\xi u+F_{mem}=0. \end{array}$$ (7)

In Eq (7), the first term describes the strain rate tensor where *η*(*ϕ*) is the viscosity. It is defined as *η*(*ϕ*) = *η*_*m*_ *m*4*ϕ*(1 − *ϕ*)+ *η*_*c*_ *ϕ*, where *m* is the non-dimensionalized variable for actomyosin concentration [*M*], 4*ϕ*(1 − *ϕ*) is approximately 1 in the interface where the cortex resides, *η*_*m*_ is the viscosity coefficient around the cortex region and *η*_*c*_ is the viscosity coefficient for the cytoplasm. We assume that the viscosity around the cortex is proportional to actomyosin concentration, and the viscosity coefficient in the cortex is greater than the one in the cytoplasm (*η*_*m*_ > *η*_*c*_).

The second term, *ξ* **u**, is the hydrodynamic drag [25]. We consider the hydrodynamic drag force since the extra-embryonic matrix (EEM), the space between membrane and eggshell, is fluid-filled [5, 26].

The last term in Eq (7), *F*_*mem*_, is the sum of forces by the membrane/cortex. Since we assume cortex and membrane to be a single entity, the forces resulting from membrane deformations and cortex contractions are considered together as follows:

$$\begin{array}{c} F_{mem}=F_{tension}+F_{bending}+F_{eggshell}+F_{volume}+F_{actomyo}. \end{array}$$ (8)

In particular, the first four forces can be derived from the surface free energy *E* by taking the variational derivative of the energy functional $-\frac{\delta E(\phi )}{\delta \phi }$. In the following we describe the individual forces in detail.

#### Force due to elastic bending *F*_*elastic*_

The Canham-Helfrich bending energy for the surface which represents the resistance to bending is given by

$$\int_{\Omega_{s}}\frac{k}{2}H^{2}\;ds,$$

where *k* is the bending rigidity and *H* is the mean curvature. The phase field representation of this elastic energy is defined by (see [18, 20])

$$E_{bending}=\int_{\Omega }\frac{\kappa }{2\epsilon }[\epsilon \nabla^{2}\phi -\frac{1}{\epsilon }G^{\prime }{)}^{2}]dx,$$

where *ϵ* is the transition parameter. The bending force on the membrane resulting from the bending energy is defined as follows [18, 25]:

$$F_{bending}=\kappa \epsilon (\nabla^{2}-\frac{G^{\prime \prime }}{\epsilon^{2}})(\nabla^{2}\phi -\frac{G^{\prime }}{\epsilon^{2}})\nabla \phi .$$

#### Force due to surface tension *F*_*tension*_

Here we consider the surface tension from both the membrane and the cortex. The global membrane surface tension is denoted by *γ*; the surface tension due to the cortex is inhomogeneous and linearly proportional to the actomyosin concentration *m* (non-dimensionalized variable for [*M*]) [1]. Therefore, the overall surface tension is written as *γ* + *c*_*T*_*m*, where *c*_*T*_ is a constant, and the energy is

$$E_{tension}=\int_{\Omega }\left(\gamma +c_{T}m\right)(\frac{\epsilon }{2}{\left|\nabla \phi \right|}^{2}+\frac{G}{\epsilon })dx,$$

with its corresponding force [18, 25]

$$F_{tension}=-\left(\gamma +c_{T}m\right)(\epsilon \nabla^{2}\phi -\frac{G^{\prime }}{\epsilon })\nabla \phi .$$

#### Force due to volume constraint *F*_*volume*_

The volume of the cell is assumed to be almost constant during the establishment phase. Since we are using a two-dimensional model, the volume constraint is translated to the area constraint, which is associated with the term

$$E_{volume}=M_{1}{\left(\int_{\Omega }\phi \;dx-\alpha \right)}^{2}.$$

Here, *α* = ∫_Ω_ *ϕ*(**x**, 0)*d***x** is the initial area of the interior of the cell on the 2D plane. This energy functional penalizes the system with coefficient *M*_1_: if the volume is changed, this constraint will drive it to *α*. The force generated by this energy term is [18]:

$$F_{volume}=M_{1}(\int_{\Omega }\phi \;dx-\alpha )\frac{\nabla \phi }{\left|\nabla \phi \right|}.$$

#### Force due to eggshell constraint *F*_*shell*_

The eggshell is a rigid body that surrounds the embryo during early development of *C. elegans* and constrains its movement and shape. An energy functional

$$E_{shell}=M_{s}\int_{\Omega }{\left(\phi \phi_{s}\right)}^{2}dx$$

is defined to penalize the system if the cell tries to pass through the eggshell. Here *M*_*s*_ is the penalty coefficient and *ϕ*_*s*_ is a fixed phase function that defines the interior (*ϕ*_*s*_ = 0) and exterior (*ϕ*_*s*_ > 0) of the eggshell. The term *E*_*shell*_ will be positive if the cell passes through the eggshell and remains zero if the cell resides within the eggshell. The force generated by this energy term is:

$$F_{eggshell}=M_{s}\left(\phi \phi_{s}^{2}\right)\frac{\nabla \phi }{\left|\nabla \phi \right|}.$$

#### Force due to actomyosin contractility *F*_*actomyo*_

Because the cortex is a thin layer beneath the membrane, we treat the contractions in the cortex as a surface force. We model the actomyosin contraction force as a force normal to the interface of *ϕ*

$$\begin{array}{c} F_{actomyo}=(c_{m}m+c_{g}\left|\nabla m|\right)\frac{\nabla \phi }{\left|\nabla \phi \right|}. \end{array}$$

The force is composed of two parts. The first part is the *c*_*m*_ term which describes the actomyosin contractility in cross-linked form. This contractility is isotropic and assumed to be proportional to the actomyosin concentration, therefore high in the anterior and low in the posterior (see Fig 1D). In the remainder of the paper, we will call this force “cross-linked actomyosin contractility”. The second part, the *c*_*g*_ term, describes the contractility due to aligned actomyosin which is described below. During the establishment phase, the pseudocleavage furrow forms at the transition zone between high and low actomyosin concentrations [27] and moves simultaneously with the edge of the anterior protein domain towards the anterior pole as the actomyosin cap regresses to the anterior [10, 14]. The formation of the furrow can be attributed to a change in actomyosin concentration because it is located in the transition zone of high to low actomyosin. In a recent study [27], it was shown that the change of the flow velocity of proteins from high to low along the anterior-posterior axis leads to a compression which aligns the filaments circumferentially. This alignment creates a higher tension around the furrow, as illustrated in Fig 1D. We represent this tension as an inward force, referred to as “aligned actomyosin contractility”. The magnitude of this force is approximated by *c*_*g*_|∇*m*| to reflect the fact that it only acts around the pseudocleavage furrow.

### Model setup and parameters

The domain of our model is taken to be [−45 μm, 45 μm] × [−45 μm, 45 μm] on the *x* − *y* plane. Considering the size of an early *C. elegans* embryo which is approximately 50 μm in length and 30 μm in diameter, the initial phase field function *ϕ* is taken to be an ellipse with radii 15 and 25, and is defined by:

$$\begin{array}{c} \phi \left(x,y\right)=0.5(\tanh(\frac{1-\sqrt{{\left(\frac{x}{15}\right)}^{2}+{\left(\frac{y}{25}\right)}^{2}}}{0.025\epsilon })+1), \end{array}$$ (9)

where *ϵ* is the parameter that scales the width of the interface in the phase field function *ϕ*, and is taken to be 2 in this work. The boundary conditions for *ϕ* are taken to be periodic boundary conditions for the ease of using Fourier transform in computation.

Initially, the concentration of anterior Par proteins is high in the anterior part of the cell and low in the posterior part, and the posterior Par proteins have a reciprocal distribution. To determine initial conditions of the species, the bistable steady-state solutions of model (1)–(5) with high and low concentrations are found for all species. The initial distributions of [*A*_*m*_], [*A*_*sd*_], [*A*_*dd*_] and [*P*] are defined on the *x* − *y* plane and are of the form $c_{1}(0.5-0.5\tanh(\frac{-y-K_{0}}{0.5\epsilon }))+c_{2}$, where *K*_0_ determines the location of the initial interface between the high and low Par protein concentrations, and *c*_1_ and *c*_2_ are the high (low) and low (high) base concentrations. We chose the initial interface between high and low anterior Par proteins to be close to the posterior domain to mimic the initial cue for polarity establishment. The values of *K*_0_, *c*_1_ and *c*_2_ for each species are given in S1 Table. The initial actomyosin [*M*] is assumed to be uniformly high. In this work, we take 0.7 for the initial actomyosin concentration. No-flux boundary conditions for these species are taken on the membrane.

The non-dimensionalized version of model (1)–(5) is used in the simulation, and the equations are shown in S1 Text, Eqs. (S1)-(S5). The associated parameter values listed in S1 Table. are chosen to produce desired model behavior. For the membrane evolution Eqs (6)–(8), the values of all of the parameters, their physical meanings and references are listed in S2 Table. While experimental measurements for the tension and forces are unavailable for this organism, we chose the parameters so that the results qualitatively reproduce the behavior of a pseudocleavage furrow in a wild type *C. elegans* embryo, which refers to a contracting anterior domain and an invaginating pseudocleavage furrow moving toward the anterior pole as the cell polarizes. To test how robust the model behavior is with respect to parameters, we have perturbed the some parameters in the phase field model by 20% and found that the results are very similar (see S1 Fig).

In the numerical simulations, Eqs. (S1)-(S5) are coupled to Eqs (6)–(8) alternately: given a fixed membrane profile, the reaction-diffusion equations are solved, followed by solving the phase field model using a fixed protein profile. The details of the implementation of the numerical algorithms are provided in S2 Text.

## Results

### Numerical simulations recapitulate in vivo behavior

We first wished to determine if our model could reproduce wild type behavior of the embryo during the establishment phase, as described in the Introduction and Model setup and parameters. In particular, we wish to see if our model can produce a pseudocleavage furrow, an invagination that advects with the edge of the actomyosin cap and the interface between the anterior and posterior Par proteins. This process is illustrated in Fig 1A, and a DIC image of a typical wild type *C. elegans* embryo at the end of the establishment phase, when the pseudocleavage furrow has reached the middle of the cell, is shown in Fig 1B.

**Fig 2:**
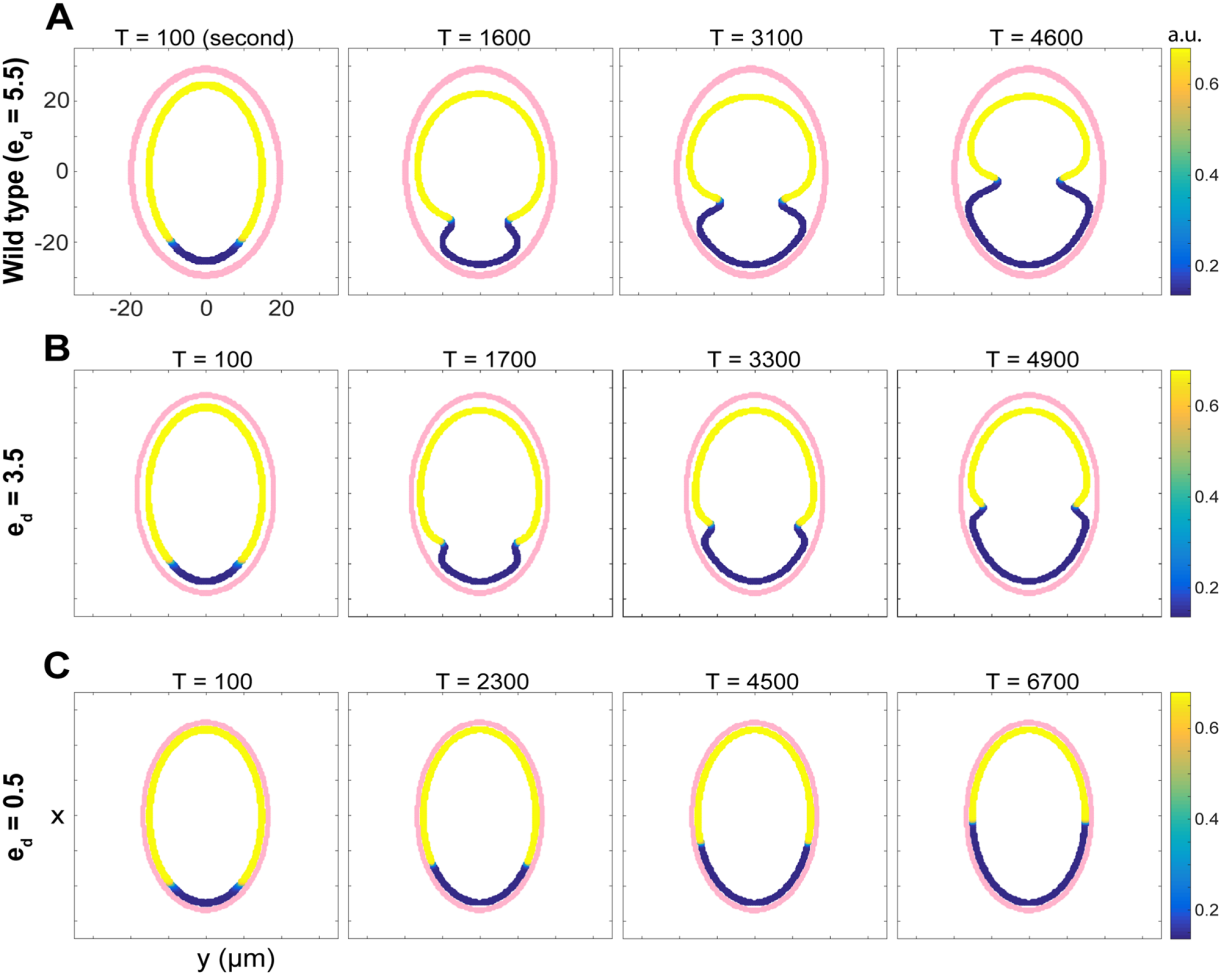
Simulations of polarization dynamics and formation of pseudocleavage furrow in the early *C. Elegans* embryo. Membrane morphology and protein dynamics obtained with distinct eggshell to membrane distances. The pink boundary represents the location of the eggshell. The cell membranes are shown with yellow-to-blue color map for the actomyosin concentrations (*m*). *e*_*d*_ is the distance between the eggshell and membrane. (A) Wild type cell with *e*_*d*_ = 5.5 μm. (B) Mutant cell with *e*_*d*_ = 3.5 μm. (C) Mutant cell with *e*_*d*_ = 0.5 μm, which mimics the case of no eggshell-to-membrane space.

Numerical simulations of our model successfully reproduce the wild type behavior, by using the estimated parameters in S1 and S2 Tables. In Fig 2A, we show the distributions of actomyosin (*m*, non-dimensionalized [*M*]) along with the deforming membrane, and its relative position with respect to the eggshell. The overall profile of the total anterior Par proteins (*a*_1_+ *a*_10_+ *a*_11_, non-dimensionalized [*A*_*m*_]+ [*A*_*sd*_]+ [*A*_*dd*_]) is almost identical to that of [*M*] qualitatively and therefore is not shown here. From Fig 2A, we observe the initial formation of a shallow invagination at the interface between anterior and posterior Par proteins occurs before *T* = 1600s (the time of the appearance of the furrow is related to the preset initial cue), and as it moves toward the anterior end, the pseudocleavage furrow deepens and reaches the middle of the cell around *T* = 4600.

Although we took a specific set of parameter in the simulation to demonstrate the wild-type behavior, our numerical experiments showed that it is possible to generate qualitatively similar behavior for a wider range of values for these parameters, which we discuss in The relationship between pseudocleavage furrow depth, time of polarity establishment and actomyosin related forces.

### The space between eggshell and membrane affects the depth of the pseudocleavage furrow and time for polarity establishment

It is known that the eggshell provides structural support for the embryo, but details about the mechanical interactions between the eggshell and the embryo are still unclear. In the experiments of [28, 29], the authors showed that mutations causing a loss of peri-vitelline space or extra-embryonic matrix (EEM), which is the space between eggshell and membrane, results in embryos lacking a pseudocleavage furrow and unable to initiate cytokinesis. This led us to investigate with our computational model how cell polarization and formation of the pseudocleavage furrow changes under different eggshell-to-membrane distances. In the following, we denote the distance between the eggshell and the membrane by *e*_*d*_. The value of *e*_*d*_ is constant throughout each simulation and does not vary as the cell undergoes shape changes. All parameters except *e*_*d*_ remained the same as in the wild-type simulations of Fig 2A.

#### Time for polarization

**Fig 3:**
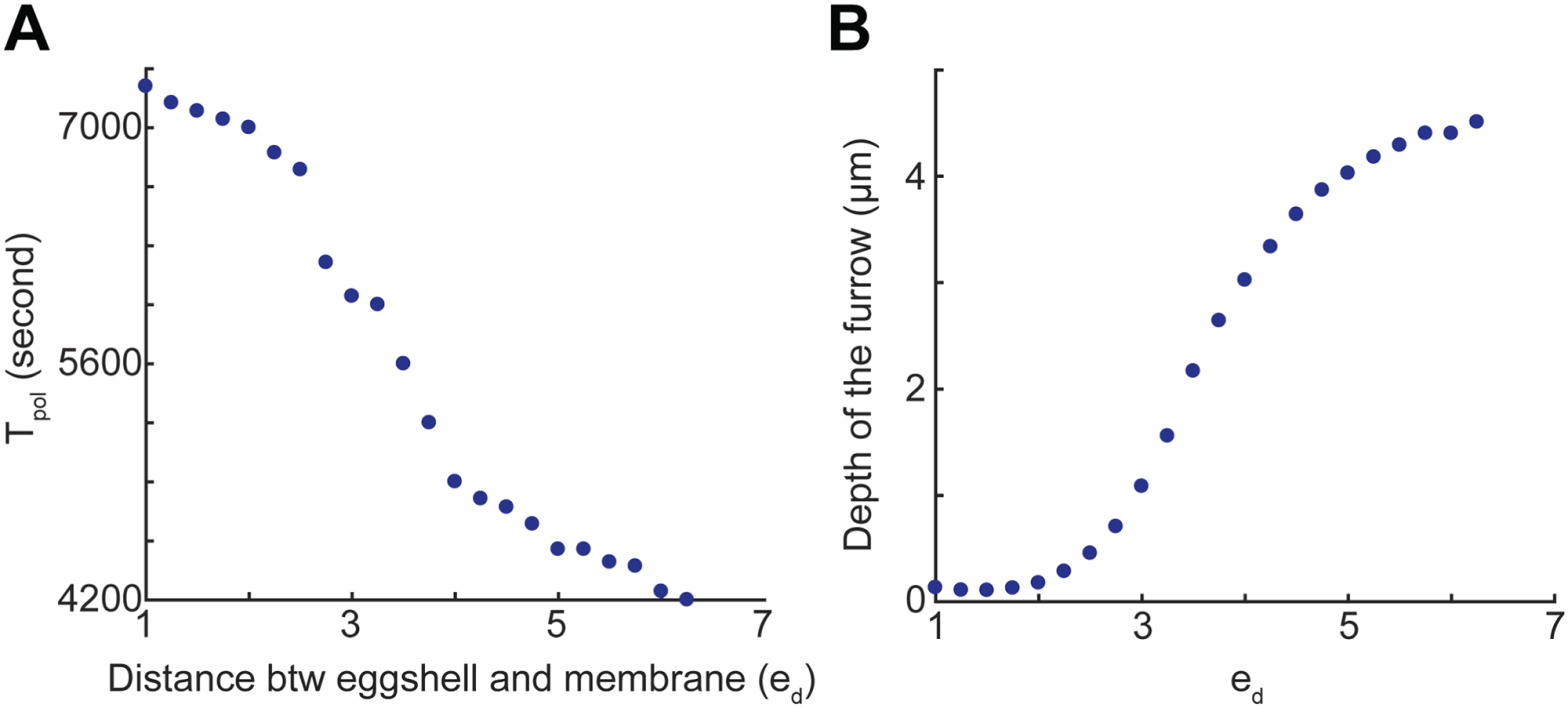
The effect of changing *e*_*d*_ on *T*_*pol*_ and the depth of the furrow. Relationship between the eggshell-to-membrane distance *e*_*d*_ to (A) the time for the furrow to reach the middle of the cell *T*_*pol*_, and (B) the depth of the pseudocleavage furrow.

As *e*_*d*_ is decreased from 5.5 μm, the parameter used in the wild type simulation in Fig 2A, to 3.5 μm, we noticed a difference in the dynamics of polarization and the depth of pseudocleavage furrow (see Fig 2B). We calculate the time from the beginning to the time that the furrow reaches the middle of the cell, which is approximately the end of the establishment phase in wild type cell, and this time duration is called *T*_*pol*_. This definition is used because it eliminates the effect of cell size, which might be changing under different conditions. It can be seen from Fig 2A and 2B that when *e*_*d*_ was decreased from 5.5 to 3.5 μm *T*_*pol*_ (in the right frames of the figures) increased from 4600 s to 4900 s. This trend is consistent when we systematically increase *e*_*d*_ from 1 to 6.25 μm: we found that *T*_*pol*_ indeed decreases as *e*_*d*_ increases, as shown in Fig 3A. In other words, when the eggshell is closer to the membrane, it takes longer for the pseudocleavage furrow to move to the middle of the cell.

#### Depth of the pseudocleavage furrow

Next, we tested how *e*_*d*_ affects the depth of the pseudocleavage furrow. The depth of furrow is defined to be the distance from the bottom of the furrow to the initial membrane prior to deformation. We measured the depths at equally spaced times (every 50 seconds) from the beginning of the simulation to the time when the furrow reached to the middle of the cell, and these values were averaged to indicate the overall furrow depth, which we call “average furrow depth” in the remainder of the paper. We can see from Fig 3B that as *e*_*d*_ gradually changes from 1 to 6.25 μm, the average depth of the pseudocleavage furrow increases from 0.11 to 4.5 μm. This is consistent with our observation in Fig 2A and 2B. However, when *e*_*d*_ is greater than 6.5 μm, the eggshell size is too big for the membrane to behave properly, which we discuss further in Geometric constraint of the eggshell is necessary for proper polarization and maintenance of cell morphology.

#### In the absence of membrane-to-eggshell space

The results shown in Fig 3B suggest that if *e*_*d*_ is close to zero, we would expect the absence of a pseudocleavage furrow and very slow polarity establishment. To test this, we performed numerical simulations with *e*_*d*_ = 0.5 μm and found that the pseudocleavage furrow does not form, as shown in Fig 2C. This result is consistent with the previous experimental findings of [28, 29], in which the removal of peri-vitelline space leads to the absence of pseudocleavage furrow. We also found that absence of the peri-vitelline space greatly extended the time to polarization in our simulation, with *T*_*pol*_ ≈ 6700, as compared to the wild type case where *T*_*pol*_ ≈ 4600 (Fig 2A).

Therefore, we concluded that the speed of the polarization is much slower when there is little or no space between the embryo and eggshell compared to the simulation with a moderate sized space. A very shallow pseudocleavage furrow was first observed when *e*_*d*_ was close to 2.5 μm. On the other hand, when *e*_*d*_ was taken to be greater than 6.5 μm, anterior contractions are too strong which leads to cell distortion, similar to simulations in which the eggshell was removed (see Geometric constraint of the eggshell is necessary for proper polarization and maintenance of cell morphology). Hence, to obtain proper polarization dynamics in the simulations, *e*_*d*_ needs to take on values between 2.5 and 6.5 μm, and this suggests that in the embryo, the presence of the pseudocleavage furrow and proper polarization may also require the space between eggshell and membrane to be in an optimal range.

### The relationship between pseudocleavage furrow depth, time of polarity establishment and actomyosin related forces

In our model, the key element that generates the cell shape changes is the actomyosin contractility forces *F*_*actomyo*_, which consists of two parts: cross-linked actomyosin contractility proportional to the actomyosin concentration (controlled by the parameter *c*_*m*_), and the aligned actomyosin contractility which is proportional to the gradient of the actomyosin concentration (controlled by *c*_*g*_). As described in Mathematical model, we differentiate these two forces by how the actomyosin are structured, although both forces are due to actomyosin contractility. We would like to investigate how modulating *c*_*g*_ and *c*_*m*_ gives rise to different membrane behaviors during the establishment phase. In particular, one of the aims is to find the ideal ranges for those parameters to generate wild-type behavior qualitatively and understand how these parameters affect the depth of the pseudocleavage furrow and the time of polarity establishment. In this section, we uniformly sampled 11 of *c*_*g*_ values and 24 of *c*_*m*_ values from the ranges *c*_*g*_ ∈ [300, 800], *c*_*m*_ ∈ [10, 240] and performed simulations for each set of (*c*_*g*_, *c*_*m*_).

#### The ratio between *c*_*g*_ and *c*_*m*_ is important in determining the depth of the pseudocleavage furrow

**Fig 4:**
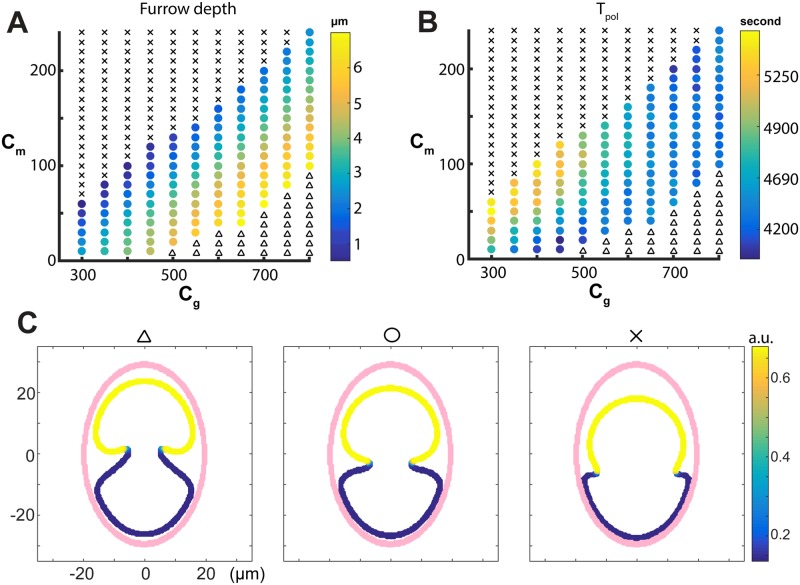
The effect of *c*_*g*_ and *c*_*m*_ on membrane morphology and protein dynamics. Different behaviors of the membrane, when different combinations of aligned actomyosin contractility (*c*_*g*_) and cross-linked actomyosin contractility (*c*_*m*_) are used. (A) The color as mapped in the color bar represents the average depths of the furrow before reaching the middle of the cell. The black triangles mark the cases in which the invagination continuously becomes deeper and oriented upwards as shown in Fig. 4C (left). The black crosses mark the cases in which the anterior part of the cell strongly contracts, leading to a much smaller anterior domain and very shallow pseudocleavage furrow which is oriented downwards as shown in Fig. 4C (right). The colored dots are the ones that successfully reproduces the qualitative wild type behavior as shown in Fig. 4C (middle). (B) The color as mapped in the color bar represents *T*_*pol*_, the time period for the furrow to reach the middle of the cell. (C) Three typical membrane behaviors corresponding to the triangles, colored dots and crosses in (A) and (B); the pink is the location of the eggshell.

In each simulation we measured the average furrow depth. Based on the simulations, we were able to classify the membrane behaviors into the following three categories: (1) the simulation successfully reproduces the qualitative wild type behavior as shown in Fig 4C (middle), that is, the membrane invaginates to form a pseudocleavage furrow and the furrow gets deeper and moves along with the interface of the anterior Par proteins; (2) the membrane invaginates and the invagination continuously becomes deeper and oriented towards the anterior end as shown in Fig 4C (left); (3) the anterior part of the cell strongly contracts, leading to a much smaller anterior domain and very shallow pseudocleavage furrow which is slightly oriented towards the posterior end as shown in Fig 4C (right). These three typical behaviors are respectively marked by black triangles, colored circles and black crosses in the *c*_*g*_ − *c*_*m*_ parameter regime of Fig 4A. Note that we only measure the depth of furrow and *T*_*pol*_ when the simulation produces wild type behavior.

It can be seen from Fig 4A that the wild type like behavior (1) is in the transition regime between (2) and (3). We observed that, with a fixed *c*_*m*_, when the alignment contractility *c*_*g*_ is very small, the anterior contraction dominates, which leads to either a very shallow furrow or behavior like Fig 4C (right). As *c*_*g*_ increases, the depth of the pseudocleavage furrow increases, up to a point where the aligned actomyosin contractility (*c*_*g*_) is sufficiently large to give rise to a very deep and anterior-oriented invagination (Fig 4C (left)). On the other hand, if the aligned actomyosin contractility *c*_*g*_ remains constant, the contraction from the anterior part of the cell increases as *c*_*m*_ increases. When *c*_*m*_ is small, the aligned actomyosin contractility generating the pseudocleavage furrow dominates and therefore the furrow is deep; as the force of *c*_*m*_ increases the furrow becomes shallower, and when *c*_*m*_ is too large, the furrow gets very shallow, and the anterior domain gets smaller due to strong contraction of the anterior domain (Fig 4C (right)). It can be inferred from Fig 4A that the regime corresponding to wild-type behavior is approximately linear, and therefore we conclude that the balance between the cross-linked actomyosin contractility (*c*_*m*_) and the aligned actomyosin contractility (*c*_*g*_) terms is critical to the formation of the pseudocleavage furrow as in wild-type shape, and it is roughly the ratio between *c*_*g*_ and *c*_*m*_ instead of the individual values that determines the depth of the furrow.

#### The anterior contractions and the depth of the pseudocleavage furrow affects the time for polarity establishment

We have previously defined the time for the polarization to reach the middle of the cell to be *T*_*pol*_ (see The space between eggshell and membrane affects the depth of the pseudocleavage furrow and time for polarity establishment). In simulations for each set of (*c_g_*, *c_m_*), we measured *T_pol_* and used colors to represent its level in Fig 4B. As seen in Fig 4B, as *c_g_* is smaller than 600 and with a fixed *c_m_*, higher aligned actomyosin contractility *c_g_* leads to faster polarity establishment. However, the trend disappears when *c_g_* becomes large than 600 because for those parameter sets, the corresponding *c_m_*’s are large and thus have a dominating effect. Combined with Fig 4A, this result suggests that, with a moderate *c_g_*, a deeper furrow has an effect in speeding up the polarity establishment. On the other hand, if the aligned actomyosin contractility (*c_g_*) is smaller than 600 and is held constant, increasing the cross-linked actomyosin contractility (*c_m_*) leads to a decreased speed of polarity establishment. Hence we conclude that for small *c_g_* the anterior contractions due to cross-linked actomyosin contractility are effective in slowing down the polarization.

### Geometric constraint of the eggshell is necessary for proper polarization and maintenance of cell morphology

We also wished to investigate how the eggshell affects Par protein polarization and cell morphology. This is difficult to study experimentally since the eggshell is essential for the survival of the early embryo. Fortunately, modeling has no such constraint.

**Fig 5:**
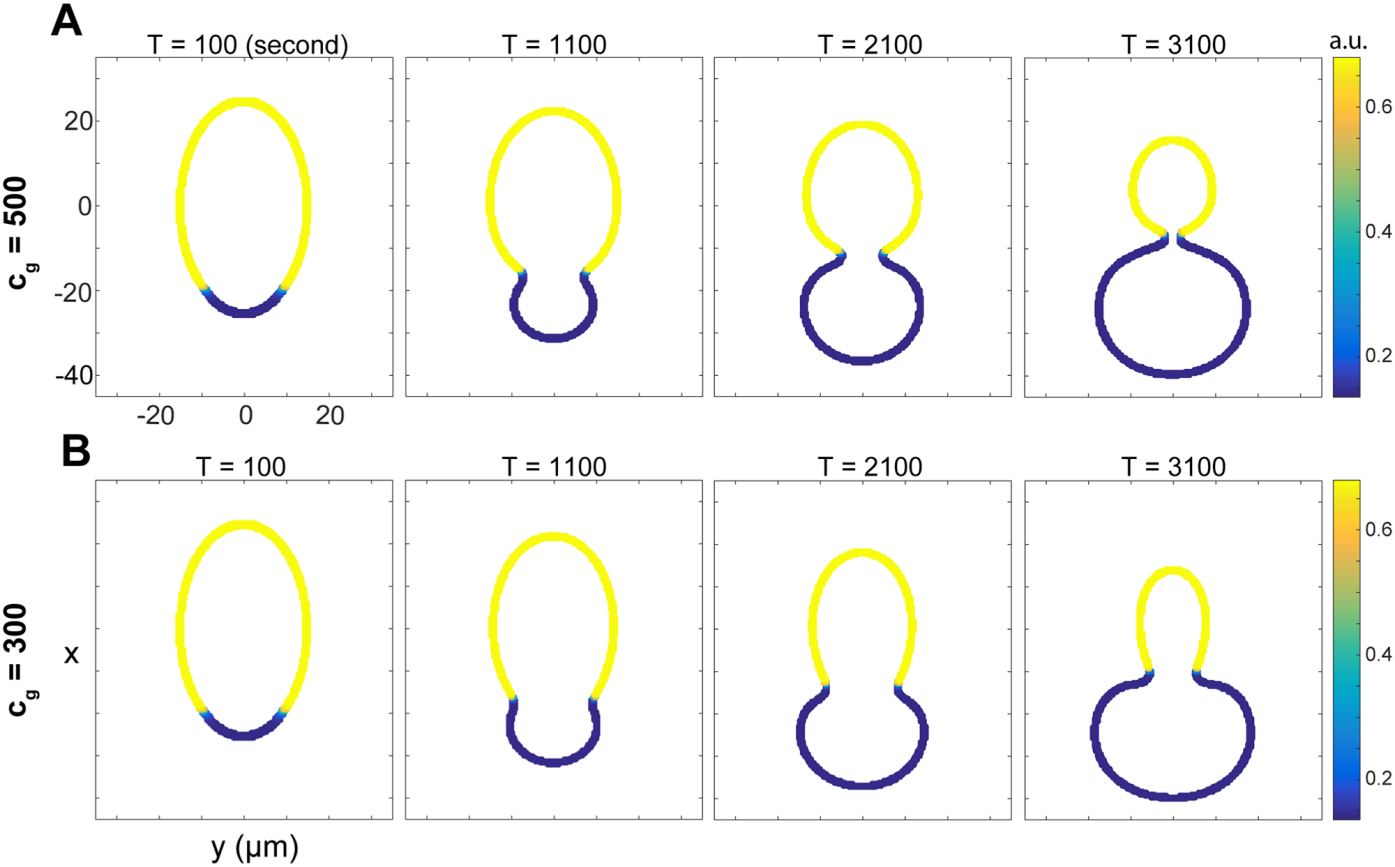
No-eggshell case. Membrane distortion when the eggshell is removed. (A) A larger aligned actomyosin contractility, with *c*_*g*_ = 500; (B) A smaller aligned actomyosin contractility, with *c*_*g*_ = 300.

Here we used the parameter set that produced the wild-type behavior in Fig 2 but take *M*_*s*_, the coefficient of the eggshell force, to be zero to model the removal of eggshell. As shown in Fig 5A, we observed that without an eggshell, the asymmetric contraction of actomyosin, which is higher in the anterior domain, led to an expanded posterior domain and a small anterior domain as the pseudocleavage furrow moves to the anterior. Moreover, the pseudocleavage furrow reaches the middle of the cell in much shorter time (2100 s) compared to the wild-type case (4600 s). This suggests that the eggshell might be significant for proper morphology as well as timing of cell polarization.

In the above numerical experiment, it was assumed that the presence of the eggshell, a nonzero value of *M*_*s*_, is the only parameter that is changed in the with/without eggshell cases. However, it is possible that the removal of the eggshell also leads to changes in the aligned actomyosin contractility (controlled by the parameter *c*_*g*_). It may be that compression between the membrane and eggshell exerts extra force, deepening the furrow ingression caused by the aligned actomyosin contractility. If this is the case, then the aligned actomyosin contractility (*c*_*g*_) will be smaller when the eggshell is removed. To test this scenario, we decrease *c*_*g*_ from 500 (wild type) to 300 and take *M*_*s*_ = 0 (no eggshell), and the results are displayed in Fig 5B. Due to the reduced *c*_*g*_, the invagination at the anterior-posterior Par protein interface disappears, that is, there is no pseudocleavage furrow. Similar to Fig 5A, the cell expands in the posterior domain, and despite the loss of furrow, the change of curvature in the cell membrane is still visible because of the differential actomyosin contraction in the anterior and posterior domains. A systematic study of varying *c*_*g*_ values in the absence of eggshell showed the continuous change in cell morphology (S2 Fig), and in all cases the time for the anterior-posterior Par protein interface to travel to the middle of the cell is significantly shorter than that for a wild-type cell.

Altogether, our simulations in Fig 5 suggest that if the eggshell is removed, the cell shape will be highly distorted, and in some cases, the pseudocleavage furrow may not form. These results suggest that the eggshell might be effective in reinforcing polarization and preserving cell morphology, and the presence of the rigid eggshell may also significantly slow down the polarization.

## Discussion

In this paper, we have used a mathematical model to investigate morphological changes of the *C. elegans* embryo during the establishment phase of its polarization process. In particular, we are interested in the role of the eggshell in formation of the pseudocleavage furrow, and the interaction between the eggshell and the asymmetric distribution of actomyosin concentration observed during polarization. Our model not only allowed us to qualitatively reproduce the experimentally observed wild-type membrane behavior and pseudocleavage furrow, but also provided biological insights into some scenarios that are unattainable with current experimental tools. Our model combines the well established phase field model to describe the morphogenesis of the cell membrane/cortex by incorporating force generated by several mechanisms: actomyosin contractility in cross-linked and aligned form, constraint from volume conservation, and constraint from the eggshell. The phase field model is coupled with protein dynamics on the cell membrane. Using our mathematical model, we have demonstrated that cell mechanics and geometry may affect protein dynamics on the cell membrane.

Previous experiments have shown that cells with mutations that eliminate the space between embryo and eggshell do not have pseudocleavage furrows and are defective in cytokinesis [28]. Our numerical simulations produced consistent results: when the space between the eggshell and membrane is eliminated, no pseudocleavage furrow was observed. Therefore the absence of a pseudocleavage furrow in mutants might have a mechanical explanation in that there is no place for a furrow ingression. The unsuccessful cytokinesis in those mutants can be explained by the lack of room for invagination of the membrane, similar to the reason for the absence of a pseudocleavage furrow. Our results also indicate that the size of the space between the eggshell and the membrane might affect the speed of protein polarization: if there is no space between eggshell and embryo, the speed of protein polarization is greatly attenuated, possibly due to the lack of a pseudocleavage furrow. If it would be experimentally possible to increase the space between the embryo and the eggshell, the model predicts that the cell will experience a shorter time to polarization and a deeper furrow relative to a wild type embryo. Our results also indicate a relationship between force generated by cross-linked actomyosin contractility and force generated by aligned actomyosin contractility, a potential area for further experimental investigation. In the numerical experiment in which the eggshell is removed, we observed that a highly asymmetric contraction of actomyosin leads to a distorted cell shape. This result suggests that the mechanical support of an eggshell plays an essential role in proper protein polarization and that if the eggshell can be removed while maintaining cell integrity, the model predicts large scale morphological changes and a small anterior domain relative to the posterior.

In Mayer et al. [30], the authors found that, in the anterior, the tension in the direction orthogonal to the anterior-posterior axis is different than the tension along the anterior-posterior axis. The anisotropy of tension increases the depth of the pseudocleavage furrow [27]. In this work, our aim was to investigate the qualitative behavior of the pseudocleavage furrow by taking advantage of a 2D model in the phase field context, therefore the simplest form of tension was considered. Another possible mechanism not being considered in this work is tension generated by compression in the tangential direction. It is possible that the eggshell increases compression, and beyond some point the cortex-membrane complex might generate the pseudocleavage furrow as a result of a buckling type of instability, leading to formation of a crease [31]. In this case, removal of the eggshell might reduce the compression, which will likely lead to the absence of a pseudocleavage furrow. However, to understand buckling behavior due to compression, we would need to include advection of the cortex in the tangential direction which cannot be tracked by the boundary tracking method we use.

In this investigation, we focussed on the large invagination formed by the pseudocleavage furrow. However, the early embryo also exhibits shallow, transient invaginations called ruffles during the establishment phase. The mechanisms behind ruffle formation are not clear. Limited change in area, contractility by actomyosin and the eggshell may have a combined effect leading to formation of creases on the surface, creating ruffles. In our model, we assumed that area can vary due to high tension in the anterior. Our model captures the overall area change in the anterior due to contraction without including the self-intersecting and invaginating area seen with ruffles. A more detailed model including cross-linking actomyosin foci might be needed to explain the mechanism behind the generation of both the pseudocleavage furrow and ruffles which we leave for future work.

## Supporting information

S1 Fig Parameter sensitivity. Some parameters in the phase field model (listed in S2 Table), including *M*_1_, *η*_*c*_, *η*_*m*_, *M*_*s*_ and *ξ* are perturbed by 20% from their original values. All the simulations are run until *T* = 4600 s.
(TIF)

S2 Fig Varying *c*_*g*_ in the absence of eggshell. (TIF)

S1 Table Parameters used in the reaction-diffusion equations in model (S1)-(S5) in S1 Text and the initial conditions of the unknown species. Parameter values for the reaction-diffusion equations are taken from [1].
(PDF)

S2 Table Parameters associated with phase field model in Eqs (6)–(8). (PDF)

S1 Text Non-dimensionalized model. (PDF)

S2 Text Numerical simulations. (PDF)
