# Supplementary figures and images for: The importance of mechanical constraints for proper polarization and psuedo-cleavage furrow generation in the early Caenorhabditis elegans embryo
Source: PLoS Comput Biol. 2018 Jul 9;14(7):e1006294. doi: 10.1371/journal.pcbi.1006294 (PMC6053242; doi:10.1371/journal.pcbi.1006294)

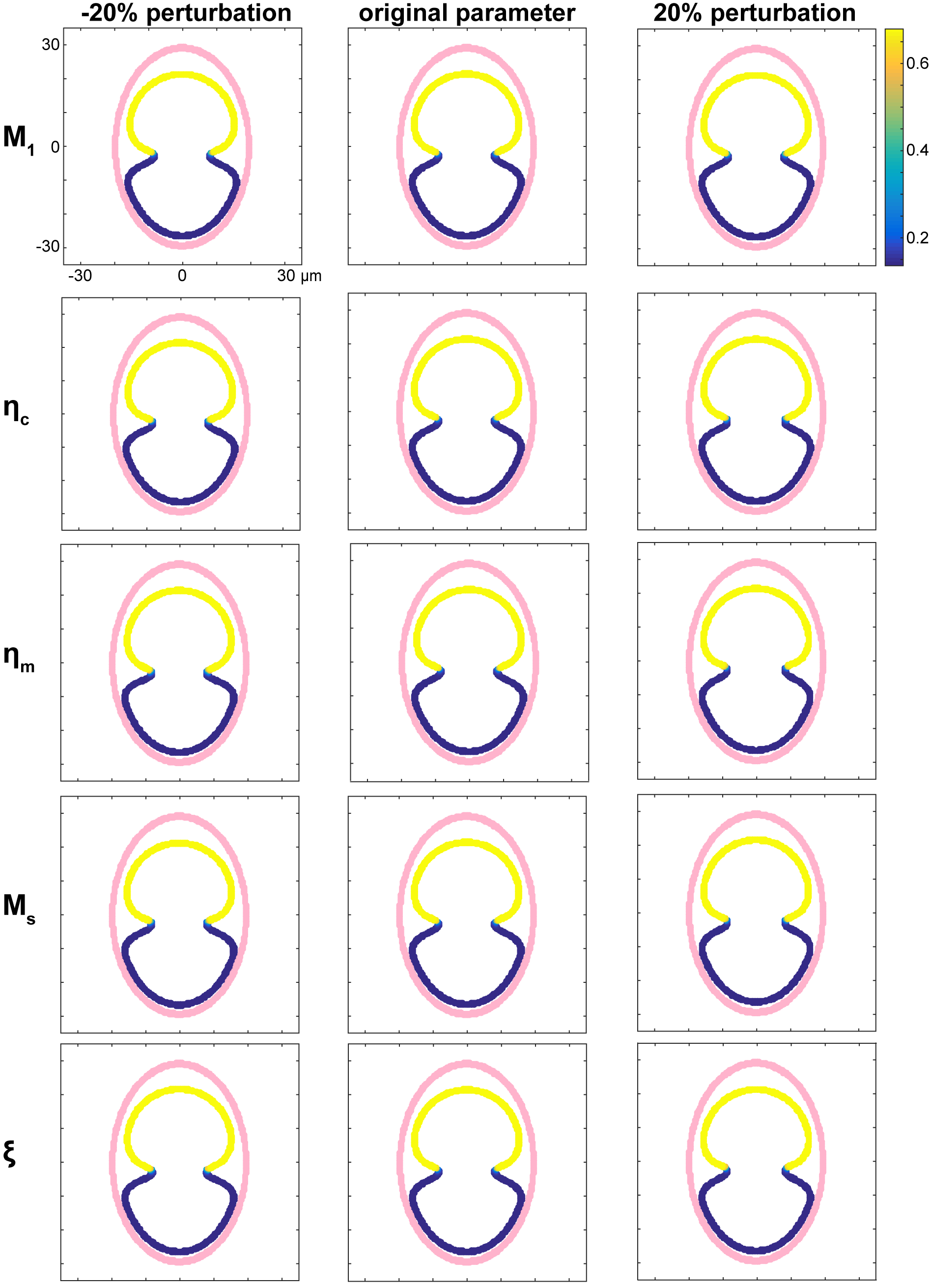

Supplement: S1 Fig — Some parameters in the phase field model (listed in S2 Table), including M1, ηc, ηm, Ms and ξ are perturbed by 20% from their original values. All the simulations are run until T = 4600 s. (TIF) [file pcbi.1006294.s001.tif]

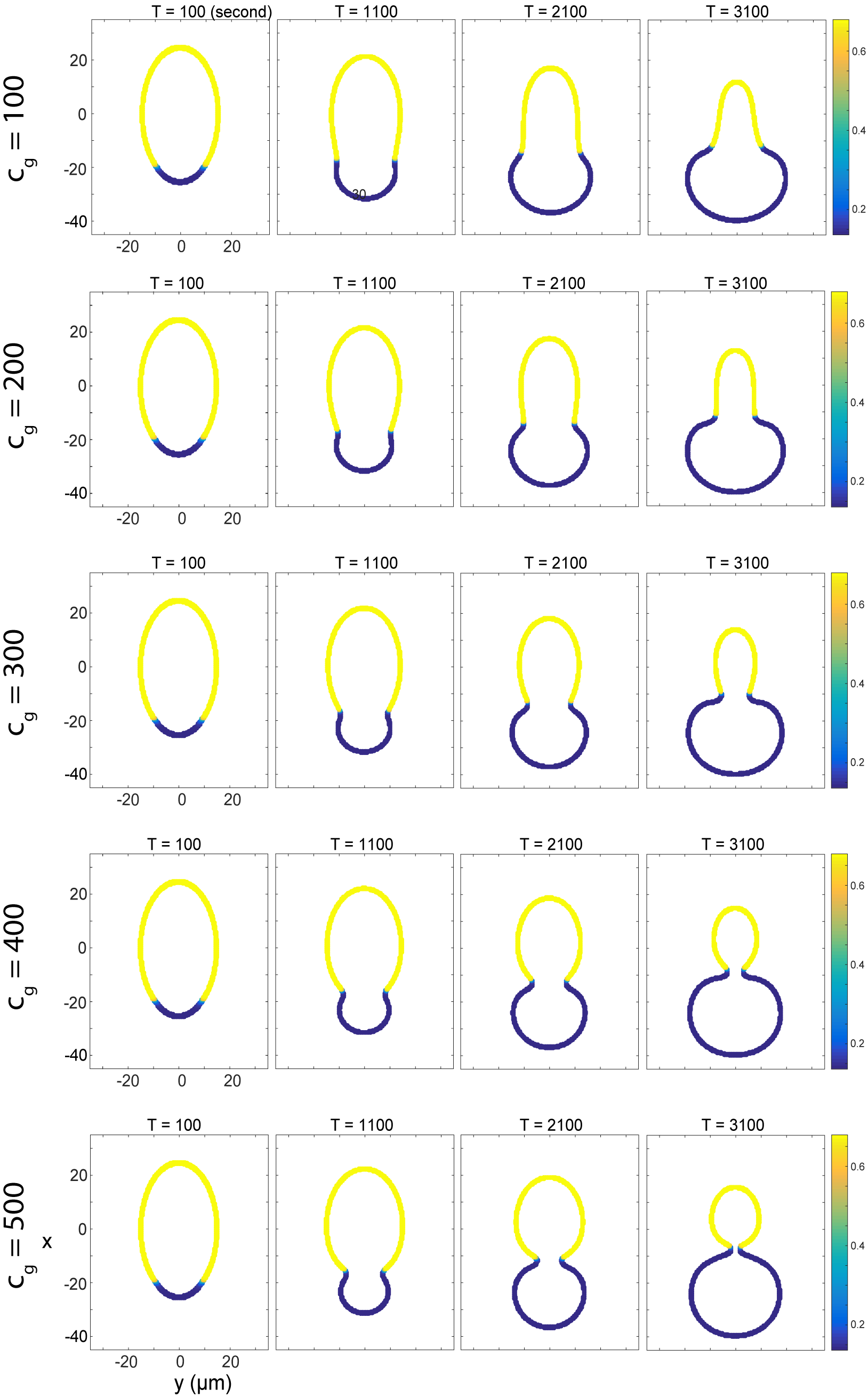

Supplement: S2 Fig — (TIF) [file pcbi.1006294.s002.tif]
