# Supplementary material for: The importance of mechanical constraints for proper polarization and psuedo-cleavage furrow generation in the early Caenorhabditis elegans embryo
Source: PLoS Comput Biol. 2018 Jul 9;14(7):e1006294. doi: 10.1371/journal.pcbi.1006294 (PMC6053242; doi:10.1371/journal.pcbi.1006294)
Supplement: S1 Table — Parameter values for the reaction-diffusion equations are taken from [1]. (PDF) [file pcbi.1006294.s003.pdf]

Parameter	Dimensional grouping	Value
β_1	k_{on}^A/k_{off}^A	0.01
β_2	$k_d^+ A_y/k_{off}^A$	4.6
β_3	$k_d^- A_y/k_{off}^A$	4.2
β_4	$r^A P_y/k_{off}^A$	0.9
β_5	$k_{on}^{A_{10}} A_{2y}/A_y k_{off}^A$	0.01
β_6	$k_{on}^{A_{11}}/k_{off}^A$	5.0
β_7	k_{on}^P/k_{off}^A	0.07
β_8	k_{off}^P/k_{off}^A	0.08
β_9	$r_P A_y/k_{off}^A$	0.7
β_{10}	k_{on}^M/k_{off}^A	0.1
β_{11}	k_P/P_y	0.1
β_{12}	k_{off}^M/k_{off}^A	0.1
D_1	$D_a/k_{off}^A L^2$	0.002
D_2	$D_p/k_{off}^A L^2$	0.0015
D_3	$D_m/k_{off}^A L^2$	0.002
μ	$\nu M_y/k_{off}^A L^2$	0.002
K_0	associated with the initial interface position of proteins	-20
c_1 for $[A_m]$	high baseline for initial $[A_m]$	0.5162
c_2 for $[A_m]$	low baseline for initial $[A_m]$	0.0164
c_1 for $[A_{sd}]$	high baseline for initial $[A_{sd}]$	0.2812
c_2 for $[A_{sd}]$	low baseline for initial $[A_{sd}]$	0.0086
c_1 for $[A_{dd}]$	high baseline for initial $[A_{dd}]$	0.4971
c_2 for $[A_{dd}]$	low baseline for initial $[A_{dd}]$	0.0069
c_1 for $[P]$	low baseline for initial $[P]$	0.0525
c_2 for $[P]$	high baseline for initial $[P]$	0.6532

S1 Table. Parameters used in the reaction-diffusion equations in model (S1)-(S5) in S1 Text and the initial conditions of the unknown species. Parameter values for the reaction-diffusion equations are taken from [1].
