# Supplementary material for: The importance of mechanical constraints for proper polarization and psuedo-cleavage furrow generation in the early Caenorhabditis elegans embryo
Source: PLoS Comput Biol. 2018 Jul 9;14(7):e1006294. doi: 10.1371/journal.pcbi.1006294 (PMC6053242; doi:10.1371/journal.pcbi.1006294)
Supplement: S2 Table — (PDF) [file pcbi.1006294.s004.pdf]

| Parameter | Description | Value | Source |
|---|---|---|---|
| $\epsilon$ | transition layer width | $2\,\mu m$ | [2] |
| $M_1$ | area constraint | $10^2\ pN/\mu m^2$ | this work |
| $M_s$ | coefficient of eggshell energy | $2 \times 10^5\ pN/\mu m^2$ | this work |
| $e_D$ | initial distance to eggshell | $5.5\,\mu m$ | this work |
| $\eta_m$ | viscosity of the cortex | $10^3\ pN\,s/\mu m$ | [2] |
| $\eta_c$ | viscosity of the cytoplasm | $10\,pN\,s/\mu m$ | this work |
| $c_m$ | coefficient of myosin contractility | 50 | this work |
| $c_g$ | coefficient of alignment stress | 500 | this work |
| $\kappa$ | bending rigidity | $20\,pN\,\mu m^2$ | [2] |
| $\gamma$ | membrane tension coefficient | $20\,pN$ | [2] |
| $c_T$ | coefficient of actomyosin tension | 600 | estimated based on [1] |
| $\Gamma$ | Phase-field relaxation parameter | $0.4\,\mu m/s$ | [2] |
| $\alpha$ | prescribed area | $1176\,\mu m^2$ | the initial area |
| $\xi$ | substrate friction coefficient | $0.5\,Pa\,s/\mu m$ | [2] |

**S2 Table. Model parameters associated with phase field model in Eqs. (6)-(8).**

.

# References


1. Tinevez J, Schulze U, Salbreux G, Roensch J, Joanny JF, Paluch E. Role of cortical tension in bleb growth. Proc Natl Acad Sci U S A. 2009;106(44):18581–18586.

2. Camley BA, Zhao Y, Li B, Levine H, Rappel WJ. Periodic migration in a physical model of cells on micropatterns. Phys Rev Lett. 2013;111(15):158102.
