# Supplementary material for: The importance of mechanical constraints for proper polarization and psuedo-cleavage furrow generation in the early Caenorhabditis elegans embryo
Source: PLoS Comput Biol. 2018 Jul 9;14(7):e1006294. doi: 10.1371/journal.pcbi.1006294 (PMC6053242; doi:10.1371/journal.pcbi.1006294)
Supplement: S1 Text — (PDF) [file pcbi.1006294.s005.pdf]

S1 Text. Non-dimensionalized model

The non-dimensionalization of model (1)-(5) is performed using the following scalings (also see [1]):

$$a_1 = \frac{A_m}{A_y},\ a_{10} = \frac{A_{sd}}{A_y},\ a_{11} = \frac{A_{dd}}{A_y},\ p = \frac{P}{P_y},\ m = \frac{M}{M_y},\ \tau = k_{off}^A t,\ x = X/L,\ y = Y/L,$$

where L is the length scale of x. To restrict the protein dynamics to the cell membrane (the diffusive interface of ϕ), the non-dimensionalized equations are coupled with the phase field function ϕ through the functions $G(\phi) = 18\phi^2(\phi-1)^2$. The resulting equations are:

$$\begin{aligned}
\frac{\partial(G(\phi)a_1)}{\partial t} &= G(\phi)(\beta_1\alpha_y - a_1 - 2\beta_2 a_1^2 + 2\beta_3 a_{11} - \beta_2\alpha_y a_1 + \beta_3 a_{10} - \beta_4 p a_1) \\
&\quad D_1\nabla_c\cdot(G(\phi)\nabla_c(a_1)) - \nabla_c\cdot(\mathbf{v}_c G(\phi)a_1), && \text{(S1)} \\
\frac{\partial(G(\phi)a_{10})}{\partial t} &= G(\phi)(\beta_5\alpha_y^2 - a_{10} + \beta_2\alpha_y a_1 - \beta_3 a_{10} - \beta_6 a_{10} + a_{11} - \beta_4 p a_{10}) \\
&\quad D_1\nabla_c\cdot(G(\phi)\nabla_c(a_{10})) - \nabla_c\cdot(\mathbf{v}_c G(\phi)a_{10}), && \text{(S2)} \\
\frac{\partial(G(\phi)a_{11})}{\partial t} &= G(\phi)(\beta_2 a_1^2 - \beta_3 a_{11} + \beta_6 a_{10} - a_{11} - 2\beta_4 p a_{11}) + D_1\nabla_c\cdot(G(\phi)\nabla_c(a_{11})) \\
&\quad -\nabla_c\cdot(\mathbf{v}_c G(\phi)a_{11}), && \text{(S3)} \\
\frac{\partial(G(\phi)p)}{\partial t} &= G(\phi)(\beta_7\rho_y - \beta_8 p - \beta_9(a_1 + a_{10} + 2a_{11})p) + D_2\nabla_c\cdot(G(\phi)\nabla_c(p)) \\
&\quad -\nabla_c\cdot(\mathbf{v}_c G(\phi)p), && \text{(S4)} \\
\frac{\partial(G(\phi)m)}{\partial t} &= G(\phi)(\beta_{10}\frac{\beta_{11}}{\beta_{11}+p} - \beta_{12}m) + D_3\nabla_c\cdot(G(\phi)\nabla_c(m)) - \nabla_c\cdot(\mathbf{v}_c G(\phi)m), && \text{(S5)}
\end{aligned}$$

where $\mathbf{v}_c = \mu\nabla m$. The variables correspond to the following species:

a_1: Cortical anterior Par protein monomers,

a_{10}: Cortical anterior singly bound dimers,

a_{11}: Cortical anterior Par protein doubly bound dimers,

p: Cortical posterior Par proteins,

m: Cortical actomyosin concentration.

The non-dimensional model and parameters are taken from [1]. The values of the parameters in the above non-dimensionalized model, and the relationship between these parameters and the parameters of the original model (1)-(5) are listed in S1 Table.
