# Supplementary material for: The importance of mechanical constraints for proper polarization and psuedo-cleavage furrow generation in the early Caenorhabditis elegans embryo
Source: PLoS Comput Biol. 2018 Jul 9;14(7):e1006294. doi: 10.1371/journal.pcbi.1006294 (PMC6053242; doi:10.1371/journal.pcbi.1006294)
Supplement: S2 Text — (PDF) [file pcbi.1006294.s006.pdf]

S2 Text. Numerical simulations

To solve the coupled reaction-diffusion equations (S1)-(S5) and phase field model (6)-(7), we alternately solve Eqs. (S1)-(S5) with a fixed phased field function and solve the phase field model (6)-(7) with a fixed protein profile. We assume that the Par protein dynamics is faster than that of the membrane evolution and therefore we chose the time step for the reaction-diffusion model to be 0.2 and the time step for Eq. (6) to be 10^{-3}.

The domain $[-45, 45] \times [-45, 45]$ is uniformly discretized into 256×256 cells. In the following we denote the numerical approximations of functions by including a subscript h. The numerical steps are described below:

1. At time $t_n = n\Delta t$, the numerical approximations of the phase filed function ϕ, velocity field $\mathbf{u}$ are denoted by $\phi_h^{(n)}$, $\mathbf{u}_h^{(n)}$, respectively.

2. To solve Eqs. (S1)-(S5), we first divide both sides by $G(\phi)$, given ϕ is fixed for a short time period. For example, Eq. (S1) becomes

$$\begin{aligned}\frac{\partial a_1}{\partial t} &= \beta_1\alpha_y - a_1 - 2\beta_2 a_1^2 + 2\beta_3 a_{11} - \beta_2\alpha_y a_1 + \beta_3 a_{10} - \beta_4 p a_1 \\ &+ \frac{1}{G(\phi)}(D_1\nabla_c \cdot (G(\phi)\nabla(a_1)) - \mu\nabla_c \cdot (G(\phi)a_1\nabla m)).\end{aligned}$$

   To avoid the denominator becoming zero, we replace $\frac{1}{G(\phi)}$ by $\frac{1}{G(\phi)+0.005}$ in the above equation. Eqs. (S2)-(S5) are dealt with similarly. We solve the reaction-diffusion system with $\phi_h^{(n)}$, and using central difference for the diffusion operator and upwind difference for the advection operator. The time step is taken to be 0.2.

3. We denote the update actomyosin from Step 2 by m_h. m_h is used as the input to calculate the actomyosin contractility force $F_{actmyo} = (c_m m_h + c_g|\nabla m_h|)\frac{\nabla\phi}{|\nabla\phi|}$, which contributes to F_{mem} in the Stokes equation (Eq. (7)). To solve the Stokes equation, we first subtract $\eta_m s\nabla^2\mathbf{u}$ from both sides of Eq. (7), where s is taken to be 8, and obtain:

$$\xi\mathbf{u} - \eta_m s\nabla^2\mathbf{u} = \nabla \cdot \Big[(\eta(\phi) - \eta_m s)\nabla\mathbf{u} + \eta(\phi)\nabla\mathbf{u}^T\Big] + F_{mem} = H(\phi, \mathbf{u}, m). \quad \text{(S1)}$$

   Note that $\eta(\phi) = \eta_m 4\phi(1-\phi) + \eta_c\phi$ is the viscosity described in the main text. As described in [25],

we use the following iterative scheme and Fourier transform to solve Eq. (S1):

$$\xi U_{k+1} - \eta_m s \nabla^2 U_{k+1} = H(\phi_h^{(n)}, U_k, m_h). \qquad k = 0, 1, .., M.$$

The initial guess U_0 is taken to be $\mathbf{u}_h^{(n)}$ and we set the solution of the iterative scheme U_M to $\mathbf{u}_h^{(n+1)}$. We set the M to be the larger one of 10 or the number obtained by using the stopping criterion

$$||U_m - U_{m-1}|| \leq 0.01||U_m||.$$

4. The final step is to update the phase field function. The phase field equation Eq. (6) can be written as

$$\frac{\partial \phi}{\partial t} = \Gamma \epsilon \nabla^2 \phi + R(\phi, \mathbf{u}),$$

where $R(\phi, \mathbf{u}) = -\mathbf{u} \cdot \nabla\phi + \Gamma(-G'(\phi)/\epsilon + c\epsilon|\nabla\phi|)$. Using a semi-implicit time discretization, we obtain the following equation

$$\phi_h^{(n+1)} - \Delta t \Gamma \epsilon \nabla^2 \phi_h^{(n+1)} = \phi_h^{(n)} + \Delta t R(\phi_h^{(n)}, \mathbf{u}_h^{(n)}),$$

from which $\phi_h^{(n+1)}$ can be solved by taking Fourier transform of both sides of the equation. We take $\Delta t = 10^{-3}$ in this work.

5. Repeat from Step 1.
